# Impressive thrombocytosis evolving in a patient with a BCR-ABL positive CML in major molecular response during dasatinib treatment unmasks an additional JAK2V617F

**DOI:** 10.1186/2162-3619-2-24

**Published:** 2013-09-05

**Authors:** Friederike Pastore, Stephanie Schneider, Oliver Christ, Wolfgang Hiddemann, Karsten Spiekermann

**Affiliations:** 1 Laboratory for Leukemia Diagnostics, Department of Internal Medicine III, University Hospital Munich – Campus Grosshadern, Munich, Germany; 2 Clinical Cooperative Group, Pathogenesis of Acute Leukemia, Helmholtz Center, Munich, Germany

**Keywords:** BCR-ABL, JAK2V617F, CML, MPS

## Abstract

We present a case of a 42-year old female with the rare diagnosis of a myeloproliferative syndrome harboring both a BCR-ABL transclocation and a JAK2V617F mutation.

Initially diagnosed with a CML, the patient underwent treatment with imatinib followed by dasatinib. Despite a major molecular response, the patient developed a thrombocytosis. Molecular analyses revealed a heterozygous JAK2V617F mutation, which was detected retrospectively in the bone marrow at the time of CML diagnosis.

This case underlines the complexity of MPS pathogenesis. For the clinician, a JAK2 mutational screening should be performed in CML patients without hematological response in the absence of BCR-ABL.

## Case presentation

In March 2005 a 42-year old woman presented with a leukocytosis of 350 G/l, a thrombocytosis of 498 G/l and an elevated lactate dehydrogenase (LDH) level of 2207 U/l. The differential blood count showed 47% neutrophils, 14% myelocytes, 11% promyelocytes, 10% myeloblasts, 5% metamyelocytes, 5% eosinophils, 5% basophils and 3% lymphocytes.

The diagnosis of a typical chronic myeloid leukemia (CML) was established after detection of an aberrant karyotype 46,XX,t(9;22)(q34;q11) in all 25 metaphases and the presence of the BCR-ABL-rearrangement (98%) in the bone marrow aspirate by FISH. The quantitative BCR-ABL/ABL ratio determined by RT-PCR was 53.0. The spleen was enlarged with 22.5 cm. The patient was classified as low risk according to Sokal [1] and Hasford [2] risk scores for survival.

After an initial treatment with hydroxyurea resulting in a rapid cytoreduction with an initial tumor lysis syndrome therapy was changed to imatinib (400 mg/day) in April 2005.

Under the treatment with imatinib the patient achieved a complete hematological remission within two months. Since cytogenetic and molecular remission was not obtained even after one year of therapy with imatinib, a mutation analysis of BCR-ABL was performed in October 2006, which revealed no evidence for point mutations within the BCR-ABL kinase domain. After an initial decrease, the BCR-ABL/ABL ratio continuously increased from 3.4 to 33.3 in February 2009. The patient was recommended to increase the dose of imatinib, which she declined.

**Figure 1:**
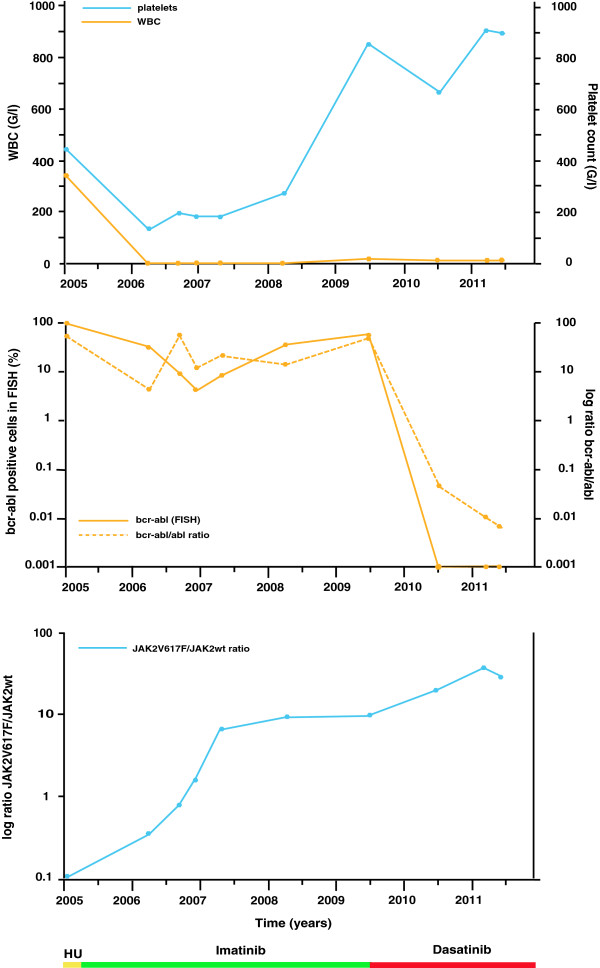
Summary of blood counts, molecular analyses and cytoreductive therapy in a patient with a CML with JAK2V617F clonal evolution under treatment with imatinib and dasatinib.

In June 2009, the patient developed a hematological relapse of the CML with leukocytosis (24 G/l), thrombocytosis (853 G/l) and elevated LDH level (290 U/l). Interphase FISH detected 59% BCR-ABL positive cells and the BCR-ABL/ABL ratio was markedly elevated (48.13). Therapy was changed to a second line regimen with dasatinib (Figure 1).

Within three months of therapy with dasatinib, a hematological response with normalization of the white blood count (WBC) and platelet count, as well as a complete cytogenetic (no BCR-ABL positive nuclei in FISH) and a partial molecular remission (BCR-ABL/ABL ratio dropped to 0.6) was recorded.

A major molecular response, with a normal karyotype and a BCR-ABL/ABL ratio <0.1 (0.04) in the quantitative RT-PCR was diagnosed in October 2010.

Until March 2013 repeated molecular and cytogenetic analyses showed a constant complete cytogenetic and major molecular remission.

Despite the absence of the Philadelphia chromosome, the patient developed and retained a marked thrombocytosis and elevated LDH levels again since February 2010.

Therefore, a JAK2-V617F mutation analysis was performed in October 2010, which demonstrated a heterozygous V617F [3].

The amount of the mutated JAK2-allel was quantified with the MutaQuant™ kit from Ipsogene. Retrospective analyses of gDNA from peripheral blood or bone marrow samples from the first diagnosis until June 2010 revealed that the JAK2 mutant allel had already been present in the bone marrow at first diagnosis at a very low level (ratio JAK2V617F/JAK2wt : 0.122).

The amount of JAK2 mutant allel was constant in 2006 and showed a rapid increase in 2007 (from 1.091 in January to 6.754 in May) under treatment with imatinib. In June 2006, when the CML relapse was diagnosed and therapy was changed to dasatinib, the JAK2V617F/JAK2wt ratio was 9.9 and further increased since then to 30.3 in May 2011. Thus, both, BCR-ABL and JAK2V617F coexisted simultaneously. Whereas leucocytosis was due to genetic abnormalities, persistant thrombocytosis was mainly caused by the JAK2V617F.

## Conclusion and discussion

We present a patient with a BCR-ABL positive CML who achieved a major molecular response after second line treatment with dasatinib. Occurrence of an impressive thrombocytosis and elevated LDH levels in the absence of BCR-ABL led to the diagnosis of an additional JAK2V617-positive clone. Retrospectively, the JAK2V617F mutant was already detectable at a very low level simultaneously to BCR-ABL at first diagnosis of CML. This is a rare case unmasking a JAK2V617F-positive clone in a patient with a BCR-ABL positive CML.

JAK2 is a tyrosine kinase that plays an important role in the signalling pathways of myeloid hematopoietic cells. A single acquired activating point mutation in JAK2 (V617F) occurs in the majority of BCR-ABL negative MPS such as ET, PV and OMF [4-7], but is only rarely found in BCR-ABL positive CML [8,9].

The hypothesis, that BCR-ABL and JAK2V617F are mutually exclusive [8] has been disproved in the last few years.

There have been reports about patients with a previous history of a JAK2V617F positive polycythemia vera (PV) [10-12], ET [13] or osteomyelofibrosis (OMF) [14] that developed a BCR-ABL positive CML and other patients that suffered a CML who developed features of a PV [15,16], an ET [17] or an OMF [18,19].

The JAK2V617F mutation was shown to precede the acquisition of BCR-ABL [10,14], co-occur with [16,19,20] and also succeed [15,21] the BCR-ABL fusion transcript.

In the published literature data are still controversial concerning the aspect if Philadelphia chromosome negative MPS and CML develop as separate diseases originating from different stem cells, or if these entities evolve from a mutual cell of origin by the acquisition of JAK2V617F and the BCR-ABL fusion gene at different time points.

Some authors favor the hypothesis, that a single sub-clone of progenitor cells carrying a JAK2V617F successively acquires the BCR-ABL translocation [10]. Those cells acquire a proliferative advantage, but disappear when exposed to imatinib. In contrast, the clone harboring JAK2V617F only will not be sensitive to imatinib.

Other models postulate that a JAK2V617F and the BCR-ABL fusion derive independently parallel from susceptible polyclonal stem cells [15,22-24] or might be preceeded by another independent genetic hit (founder mutation) which predisposes to their acquisition [18].

In our patient, at initial diagnosis a JAK2V617F was detectable at a very low level whereas the BCR-ABL clone was already 100% of the CML clone. Considering the mere frequencies this suggests that the two mutations might have arisen independently in different bone marrow cells.

Nevertheless, applying the hypothesis of Bocchia et al. [10] this does not exclude the possibility that the BCR-ABL positive clone might have represented a sub-clone of JAK2V617F mutated cells which had gained a growth advantage.

Under therapy with imatinib the JAK2 mutant level increased suggesting a growth advantage of JAK2V617F positive/BCR-ABL negative cells in relation to JAK2V617F positive/BCR-ABL positive cells which might have been at least to some degree antagonized by imatinib. These observations are in line with the fact that CML therapy with imatinib was shown to reveal Philadelphia chromosome negative clonal disorders in more than 10% of CML patients [25].

Finally, therapy with dasatinib might have eliminated the JAK2F617F-positive/ BCR-ABL positive clone. The suppression of the BCR-ABL clone might have facilitated growth of the JAK2V617F-positive cells without the BCR-ABL fusion that were unaffected by dasatinib. This theory is supported by continuously rising levels of the JAK2V617F/JAK2wt ratio and progressive thrombocytosis and leukocytosis in our patient. This is in line with the so called “gene dosis hypothesis” meaning that JAK2V617F expression level correlate with the number of involved cell lines and thus the type of disease (low level - > ET; high level - > PV) [26,27]. The increase of platelets to > 1000 G/l was followed by an increase of leukocytes.

Our data suggest the co-occurence of different clones carrying either JAK2V617F or BCR-ABL. Yet it does not exclude the possibility of BCR-ABL/JAK2V617F double-positive sub-clones.

According to our hypothesis, the BCR-ABL clone which showed an initial clonal pre-dominance was successfully reduced by treatment with dasatinib which unmasked clinical features attributed to JAK2V617F-positive disease.

These results represent the complex pathogenesis of MPS and demonstrate the possibility of overlaps or coexistences between the BCR-ABL positive and negative MPS. For the clinician, we recommend to analyze the JAK2 mutation status in patients with an established diagnosis of CML who develop myeloproliferation and/or disease progression despite molecular remission of the CML.

## Consent

Written informed consent was obtained from the patient for publication of this Case report and any accompanying images. A copy of the written consent is available for review by the Editor-in-Chief of this journal.

## Competing interests

The authors declare that they have no competing interests.

## Authors’ contributions

FP wrote the paper, performed analysis of data and interpretation of data. SS performed the cytogenetic and molecular analyses, analysis of data and interpretation of data. OC was involved in the medical care of the patient, performed analysis of data and interpretation of data. WH designed the study and performed interpretation of data. KS designed the study and performed interpretation of data. All authors read and approved the final manuscript.
